# Preparation and characterization of beta-glucan particles containing a payload of nanoembedded rifabutin for enhanced targeted delivery to macrophages

**DOI:** 10.17179/excli2016-804

**Published:** 2017-03-07

**Authors:** Tarun K. Upadhyay, Nida Fatima, Deepak Sharma, V. Saravanakumar, Rolee Sharma

**Affiliations:** 1 Department of Biosciences, Integral University, Lucknow, 226026, India; 2 Pharmaceutics Division, CSIR-Central Drug Research Institute, Lucknow, 226031, India

**Keywords:** glucan particles, targeted drug delivery, spray drying, macrophage, rifabutin, phagocytosis

## Abstract

β-glucan particles (GP) are polymeric carbohydrates, mainly found as components of cell wall fungi, yeast, bacteria and also in cereals such as barley and oat, and have been recently shown to have application in macrophage-targeted drug delivery. The aim of this study was to prepare and characterize GP containing a large payload of Rifabutin (RB), an anti-tuberculosis drug effective against MDR-TB at lower MIC than Rifampicin. GP were prepared from yeast cells by acidic and alkaline extraction were either spray dried or lyophilized, prior to RB loading and alginate sealing. The FTIR and ^13^C-NMR spectra of the GP confirmed a β-(1→3) linked glucan structure, with a triple-helical conformation. The spray dried GP exhibited better characteristics in terms of uniformity, size range (2.9 to 6.1 µm) and more than 75 % particles were below 3.5 μm. The RP-HPLC analysis of spray dried GP revealed drug entrapment and drug loading up to 81.46 ± 4.9 % and ~40.5 ± 1.9 %, respectively, as compared to those dried by lyophilization. Electron microscopy showed nearly spherical and porous nature of GP, and the presence of drug 'nanoprecipitates' filling the pore spaces. The formulation showed adequate thermal stability for pharmaceutical application. The particles were readily phagocytosed by macrophage(s) within 5 min of exposure. Drug release occurred in a sustained manner via diffusion, as the release kinetics best fit for drug release was obtained using Higuchi's equation. Thus, the spray dried GP-based-formulation technology holds promise for enhanced targeted delivery of anti-TB drug(s) to macrophage within a therapeutic window for the clearance of intracellular bacteria*.*

## Introduction

Tuberculosis (TB) still causes ~1.5 million deaths worldwide, amounting to over 95 % of the deaths in low and middle-income countries (WHO, Tuberculosis fact sheet, October 2014[41]). The 'Directly Observed Treatment, Short Course' chemotherapy (DOTS) treatment regimen is long and arduous, making patient compliance difficult and leading to the rapidly expanding problem of drug resistance. Macrophage serve as the primary cells where the Mycobacteria invade and actively replicate within maturation arrested phagosomes (Hestvik et al., 2005[17]). The targeted delivery of anti-TB drugs to these cells, therefore, forms an effective therapeutic approach against TB. Recent researches by several investigators on drug-loaded particulate delivery systems such as microparticles (Suarez et al., 2001[38]; Sen et al., 2003[31]; Verma et al., 2013[40]; Goyal et al., 2015[13]), nanoparticles (Zahoor et al., 2005[44]), liposomes (Gursoy et al., 2004[15]) etc. have shown positive results for TB therapy. Such particles are rapidly phagocytosed by Mϕ (Hirota et al., 2010[18]), and lead to the development of high intracellular drug concentrations (Sharma et al., 2001[33]), Mϕ activation (Sharma et al., 2007[32]; Yadav et al., 2009[42]; Sharma et al., 2011[34]) and significant enhancement in the anti-microbial efficacy of the loaded drugs (Sen et al., 2003[31]; Quenelle et al., 1999[29]).

Particulate delivery systems offer advantages such as improved bioavailability, particularly of hydrophobic drugs; extended drug half life; the possibility of controlled release and low drug wastage. However, the development of effective drug delivery system(s) presents multiple challenges, such as issues of drug solubility, selective targeting, *in vivo* stability and toxicity. Several delivery systems have been developed for targeting drug molecules specifically to macrophages based on the pattern-recognition receptors (PRR) on their surface (Etzerodt et al., 2012[10]; Tiwari et al., 2011[39]; Zhao et al., 2013[46]; Zhu et al., 2013[47]). Targeted delivery helps to reduce drug dose(s) and dosing frequencies, drug toxicity and side effects, and thus hold promise for improved patient compliance. However, the expression of PRR ligands on carrier surface involves intricate procedures. 

β-Glucan particles (GP) extracted from the cell walls of baker's yeast are porous, 1-4 µm spherical shells composed primarily of β-1,3/1,6-D-glucan (Soto et al.*,* 2010[36], 2012[37]). β-1,3 glucans serve as pathogen associated molecular patterns (PAMPs), that engage dectin-1 receptors on Mϕ, leading to their activation and the induction of an immune response (Herre et al.*,* 2004[16]; Batbayar et al.*,* 2012[2]). The hollow cavity within the GP allows adsorption and encapsulation of payload molecules, and thus GP have been used as effective delivery vehicle(s) to target Mϕ in the recent years (Soto et al.*,* 2010[36]; Yu et al.*,* 2015[43]). β-Glucan exists in single or triple helical conformation in solution, of which the triple helical configuration glucans have been reported as powerful immunomodulators (Falch et al.*,* 2000[11]). 

One significant aspect of drug targeting is to deliver a sufficiently large amount of drug to the target cells, so that the resulting intracellular drug concentrations are maintained within the therapeutic window (above MIC) over a period of time, and ensure complete killing of mycobacteria residing in Mϕ. Recently, an anti-TB drug, rifampicin (Rif) had been encapsulated within GP, and was found effective in reducing *M. tb.* burden within infected bone marrow Mϕ (Soto et al.*,* 2010[36]). These particles contained payloads ranging from 10 to 33 % w/w Rif/GP, and displayed rapid release of the drug through the polymer matrix within the first 24 h. The 10 % w/w GP-Rif-Alg formulation contained ~0.2 pg Rif/glucan particle, representing only 80 % of the MIC of free Rif (0.25 μg/mL). However, these particles showed an 80 to 90 % inhibition of CFU recovered from macrophage lysates after 24 or 72 h of incubation, respectively. Low drug loading and lack of control over timed release implies a need for repeated administration, inability to address the non-compliance problem and thus, a risk for development of drug resistance. It is thus highly desirable to develop GP formulations with a larger proportion of the incorporated drug payload, for their use as efficient macrophage-targeting drug delivery systems against TB. Moreover, while the standard TB therapy recommends the use of rifamycins as part of the DOTS short course therapy (Boogaard et al.*,* 2009[4]), the increasing resistance to Rif emphasizes the need for new effective drug(s) against TB. The anti-TB drug Rifabutin (RB) was thus selected for incorporation into GP owing to its higher efficacy (lower MIC range), relevance in MDR-TB treatment (Jo et al.*,* 2013[23]) and longer half-life than Rif (Dickinson et al.*,* 1987[8]; Blaschke and Skinner, 1996[3]). 

In continuation with the ongoing research on polymeric formulations against TB, the prime objective of the present investigation was to prepare biocompatible GP-based formulations containing a high-payload of the anti-TB drug, RB for Mϕ targeted drug delivery. Yeast derived GP were loaded with RB and characterized for their size, morphology, drug loading, drug release kinetics and *in vitro* uptake by Mϕ. In addition, analytical techniques were used to evaluate the chemical composition and thermal stability of these formulations.

## Materials and Methods

### Cell culture and maintenance

J774A.1 mouse Macrophage cell line was purchased from National Centre for Cell Science (NCCS, Pune). All the cell culture products DMEM, FBS, Antibiotic and Antimycotic solution were purchased from Gibco^TM^ (Thermo Fisher Scientific). Culture flask and 6-well plates were from Nunc (Thermo Scientific).

### Reagents and solutions

Starting material dry yeast (*Saccharomyces cerevisae*) was purchased from a nearby authentic bakery (Polo Enterprises, Lucknow). Sodium alginate low viscosity (a viscosity of 2 % solution: 100-300 cP) was purchased from Sigma Aldrich. Rhodamine B (Rd B) was purchased from sigma Aldrich. All other analytical chemicals and solvents used in the study were HPLC grade purchased from Merck (Mumbai, India). All aqueous solutions were prepared from triple distilled water (TDW).

### Preparation of β-glucan particles 

GP were prepared from baker's yeast by slight modification of the alkaline and acidic extraction method described previously (Soto et al.*,* 2010[36]; Hunter et al.*,* 2002[22]), followed by either lyophilization or spray-drying. Briefly, the baker's yeast was suspended in 200 ml of 1M NaOH solution and stirred for 30 min at 60 °C. The resulting material was heated to 80 °C for 60 min and then centrifuged at 200 x g for 10 min. The sediment was washed with TDW, pH adjusted to 4-5 with HCl and incubated at 55 °C for 60 min. The alkali insoluble solids were then collected by centrifugation, washed first with TDW, and subsequently 4 times with isopropanol and twice with 40 ml acetone. To overcome the problem of particle aggregation, the semisolid GP (after acetone washing) were sonicated (Sonics, UK) for 5 min in TDW using an Ultrasonic Output frequency of 20 Kilohertz per second at 192 Watts and then homogenized (IKA^TM^) at 10,000 rpm for 10 min. The suspension was finally spray-dried using a Labultima Lu 20 Spray Dryer (Labultima, Germany) with an inlet air temperature of 110-170 °C, an outlet air temperature of 90-120 °C and an atomizer pressure of 30-100 psi. Anthrone test was carried out to confirm the carbohydrate nature of the off-white powder thus obtained. 

### Rhodamine B labelling of GP and particle counting by flow cytometry

A batch of GP was also labelled with rhodamine B isothiocyanate. Briefly, the GP were incubated with rhodamine B isothiocyanate (dissolved in dimethyl sulfoxide), in sodium carbonate buffer (0.1 M, pH 9.2) overnight at 37 °C in the dark; unreacted dye was quenched by incubation with Tris buffer (1.0 M, pH 8.3) for 30 min. The labeled GP were extensively washed with sterile water to remove color, dehydrated with absolute ethanol and acetone, and then dried in the dark at room temperature. The further processing (drug loading and alginate sealing) of these fluorescent particles was done in dark and flow cytometry analysis was performed for the counting of fluorescent dye (Rhodamine) tagged GP. 

### Incorporation of Rifabutin (RB) into GP, a first-line anti-TB drug

GP containing precipitated RB (GP-RB) were made as previously described (Soto et al.*,* 2010[36]), with a slight modification. Briefly, the dry GP were incubated in 0.2 N HCl with stock RB solution (100 mg RB per ml in 0.2 N HCl) for their hydration in a drug to polymer ratio of 1:1. To precipitate and trap RB inside the GP, Tris buffer (1M, pH 8) was added. The samples were then centrifuged twice, to remove the unprecipitated RB and buffer, frozen at -80 °C and lyophilized. The resulting 'GP-RB' formulation had a strong violet-red colored appearance.

### Alginate sealing of RB-incorporated GP

After loading the glucan particles with RB, the GP pores were sealed with calcium alginate hydrogel. 200 mg of GP-RB samples were incubated with 0.25 % sodium alginate** (**pH 8) at room temperature for 60 min and then centrifuged at 10,000 rpm for 5 min. The sealing process was repeated twice, and calcium chloride (2 %, 5µL per mg GP) was added thereafter to allow overnight cross-linking at room temperature. The resultant 'GP-RB-Alg particles' were collected by centrifugation and lyophilized to obtain an easily dispersible brick red colored powder that was stored at -20 °C. 

### Particle size and zeta potential measurement of GP

Particle size was determined by using a device (Mastersizer 2000, Malvern Instruements, UK) as described previously (Sharma et al.*,* 2001[33]), to determine if these are in the appropriate size range of 1-10 μm, for being phagocytosed by MΦ. Briefly, about 5 mg of particles were dry-mixed with an equivalent amount of sodium lauryl sulfate (SLS) and suspended in one ml Milli-Q water by vortex mixing. This slurry was added into the sampling beaker of the instrument until a laser obscuration factor of > 10 % was achieved. The average size (n = 3) in µm was determined for all formulations. The zeta potential is a measurement of the electric charge on the surface of the GP and indicates their physical stability. The zeta potential of aqueous suspensions of spray dried GP and alginate coated GP were recorded using a Malvern Zetasizer (Nano-ZS 90, Malvern Instrument, UK). These measurements were taken at least three times with independent particle batches.

### Morphological characterization of GP 

The surface morphology, shape and size of the blank and drug loaded GP were visualized by a Scanning Electron Microscope (Hitachi S3700 N) at an accelerated voltage of 15 kV. In addition, the Transmission Electron Microscope (JEOL, Japan JEM 2100) images of blank GP, drug loaded (GP-RB) particles and drug loaded-alginate sealed (GP-RB-Alg) particles were taken at the Indian Institute of Toxicology Research (IITR**)**, Lucknow to study further morphological details of these particles. 

### Thermogravimetric/Differential Thermogravimetric analysis (TG-DTA)

Thermal properties of the samples were studied by employing Thermogravimetric analyzer (TGA) and differential Thermogravimetric analysis (DTA). TGA thermograms of sodium alginate, GP particles, alginate sealed GP-RB and pure RB were recorded by employing a TGA (Model TGA-50, Perkin Elmer) in the temperature range of 50-750 °C, at a heating rate of 10° C/min in a nitrogen atmosphere.

### Validation of β-glucan by structural analysis

#### Fourier Transform Infra-red (FTIR) spectroscopy

FTIR spectroscopy was applied to analyze the spray dried, lyophilized GP isolated from the yeast cell wall by alkaline and acidic extraction method as described above. Infrared spectra of the GP were recorded with an FTIR spectrometer (Perkin Elmer Spectrum Version 10.03.06) in the range of 4000~450 cm^-1^ using KBr disk method. 

### Solid-State Nuclear Magnetic Resonance (NMR) analysis

The insolubility of particulate β-glucans posed a challenge during NMR analysis, due to the inability to detect undissolved substances. Thus a high resolution ^13^C-NMR of GP was performed in solid state to elucidate the glucan conformation.

For ss-NMR analysis, the GP powder was filled into 3.2 mm Zirconium rotor. All ssNMR spectra were recorded on 600 MHz NMR spectrometer (Advance III, BrukerBiospin, Switzerland) operating at 600.154 MHz for ^1^H, and 150.154 MHz for ^13^C frequencies with Bruker 3.2 mm DVT probe. Magic Angle Spinning (MAS) frequency was 10.0 kHz for all experiments. The spinning speed was controlled by Bruker MAS pneumatic unit within the accuracy of ± 2Hz. Recycle delay used for all experiments was 5 s. Total acquisition time for each cross polarization (CP) experiments was 0.017 sec. All ^13^C CP-MAS NMR spectra were obtained with a spectral width of 315 ppm, 1K data points and 10 k scans with a contact time of 1.0 ms (milli second).

### RB analysis in GP by HPLC

RB analysis in GP was done by slight modification of an analytical method reported by Muttil et al. (2007[27]). A Shimadzu (Japan) Class VP HPLC system with a Luna C18 column (5 m, 4.6 mm × 250 mm, Phenomenex, Torrance, USA) was used for the analysis. The mobile phase was a mixture of acetonitrile and phosphate buffer in a ratio of 45:55, which was filtered through 0.22 µm filter and degassed by sonication. RB was eluted at 7.2 min at a flow rate of 1 ml/min and was monitored using a UV detector set at 275 nm.

The drug loaded-alginate sealed GP (GP-RB-Alg) suspensions were weighed, extracted with 0.01 N HCl and then diluted in mobile phase after filtration through a 0.22 µm filter. Correspondingly, standard curves were generated in the concentration range of 5 - 40 µg RB /ml mobile phase (Figure 5B).

The drug loading (LE) and encapsulation efficiency (EE) for RB within GP were calculated as follows:

 % EE = (Experimental drug content / Theoretical drug content) x 100 (1)

 % LE= (Amount of drug in microparticles / Amount of microparticles) x 100 (2)

### In vitro drug release and kinetics

*In vitro* release of RB from spray dried GP was studied by using a USP Type II tablet dissolution test apparatus (Labindia, DISSO 2000) at a stirring speed of 100 rpm and a temperature of 37 ± 0.5° C. A dialysis membrane (Sigma-Aldrich) was cut into pieces and pre-treated as per the manufacturer instructions. Accurately weighed RB containing GP were filled in hermitically sealed dialysis bags and immersed in two kinds of release media, phosphate-buffer (pH 7.4), and acetate buffer (pH 5.2) to simulate the cytosolic pH and late phagosomal pH, respectively. The release media was supplemented with 1 % v/v tween 80 to prevent precipitation of released drug. Aliquots were withdrawn in duplicates at predetermined time intervals and fresh dissolution media were added at each time point to maintain the sink conditions. All the samples were filtered through a Millipore 0.22 µm disposable filter, and filtrate was stored at −20 °C. The drug release was measured by reverse phase HPLC method as described above. 

The intensity of absorbance was utilized to calculate the concentrations of the drug. Release profile was obtained by correlating time (h) versus drug release concentration (µg mL^-1^). The RB release data were used to study the mechanism of drug release. In attempts to study the drug release mechanism from the GP, the *in vitro* release data were fitted to various mathematical models (Zero-order, First-order, Higuchi´s model and Korsmeyer-Peppas model) to predict the drug release mechanism and kinetics. Equations and other parameters of the mathematical models are shown in Table 1(Tab. 1). Data were fitted and the linear regression of the mathematical models was evaluated using R^2^ (squared correlation coefficient).

### Cell culture and maintenance

Mouse macrophage cell line (J774A.1) was procured from the National Centre of Cell Sciences (NCCS), Pune, India. The cells were grown and maintained in Dulbecco's Modified Eagle's medium (DMEM) supplemented with 10 % FBS and 1 % antibiotic antimycotic solution, in a controlled humidified atmosphere of 5 % CO_2_ and 37 °C. 

### In vitro uptake study

Adherent 0.1 × 10^6 ^J774A.1 cells per well were seeded in a 96 well plate and incubated overnight at 37 °C in 5 % CO_2_. The cells were then exposed to fluorescent particles by replacing culture media in wells with Rhodamine-tagged GP (10 Rd-GP per cell) suspended in HBSS. After 5 min exposure, the cells were washed twice with PBS and visualized under a fluorescent microscope (Nikon eclipse TiS) with a red filter for the presence of fluorescent particles. 

### In vitro cell viability assay

The cell viability of the RB-loaded glucan GPs was determined by measuring the inhibition of cell growth using a tetrazolium dye (3-[4,5-dimethylthiazol-2-yl]-2,5-diphenyltetrazolium bromide [MTT]) assay. J774A.1 cells were seeded in 96-well plate at 1×10^6^ cells/well. Next, the cells were incubated with increasing concentrations (0-100 μg/mL) of the RB-loaded GP for 24 h in CO_2_ incubator at 37 °C. Then, 10 μL MTT solution (5.0 mg/mL) was added to each well. The plates were incubated with MTT for an additional 4 h at 37 °C. After that supernatant was removed, 100 μL DMSO was added to fully dissolve the crystals. Absorbance at 490 nm, which is related to the number of metabolically active cells, was measured using a microplate reader (EXL-800; BioTek Instruments). The cell viability was calculated using the following formula: 

Cell viability (%) = (Abs. sample / Abs. control) × 100. 

The viability of untreated controls was normalized to 100 %. % Cytotoxicity = 100 - % cell viability.

### Data analysis

All data were expressed as the mean ± standard deviation of the mean from three separate experiments. Statistical analyses were conducted using Origin 6.0 and Instat software. Results of the *in vitro *drug release study and cytotoxicity analysis were subjected to analysis of variance (ANOVA). P values less than 0.05 were considered significant. 

## Results

### GP formulation

The initial batches of GP were prepared by alkaline and acidic extraction, followed by either homogenization or lyophilization. These particles showed conspicuous aggregation and fast sedimentation in aqueous suspension (data not shown). However, in the later spray dried batch (GP2), the particle aggregation was overcome by disrupting the particle aggregates prior to their complete drying and solidification, as described in the methods section and reported earlier by Hunter et al.*,* (2002[22]). The spray dried and homogenized particles were observed to remain in aqueous suspension for longer time periods (up to 1 h) as compared to the batches prepared without spray drying (data not shown). 

### Size and surface charge characteristics

**Figure 1:**
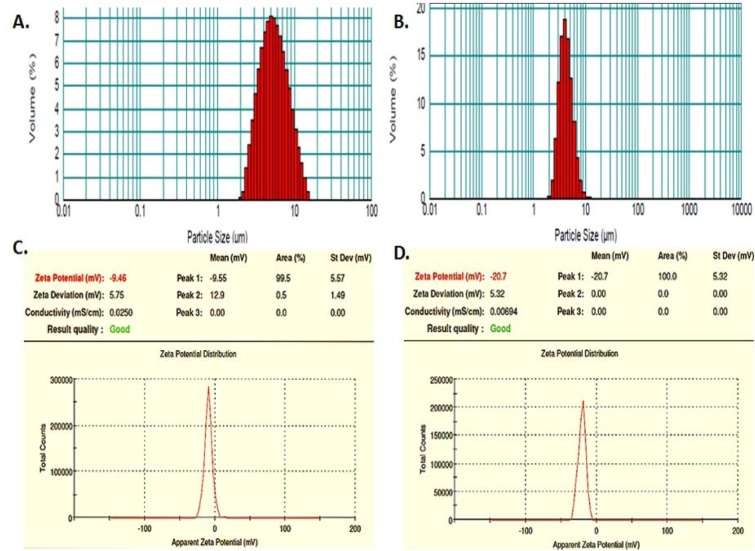
Size and surface characterization of GP. The log-normal, volume-average particle size distribution of typical batches of (A) lyophilized and (B) spray dried GP, as assessed by laser scattering. The zeta potential of (C) blank GP, and (D) RB-loaded, alginate sealed GP.

The characteristics of two representative batches of blank and drug loaded yeast-derived GP formulations such as GP1- prepared by alkaline and acidic extraction method followed by lyophilization, and GP2- prepared by sonication and spray drying method. The laser scattering studies (Figure 1B(Fig. 1)) revealed a much lower size range of spray dried particles, with more than 75 % particles in the size range of 1-4 µm, as compared to approximately 32 % particles in the batch prepared without spray drying (Figure 1A(Fig. 1)). 

To detect the presence of alginate layer over the formulated GP, we compared the zeta potential of the blank GP shells (Figure 1C(Fig. 1)) with that of the alginate sealed particles (Figure 1D(Fig. 1)). The alginate coating of GP reduced the apparent zeta potential (from -9.46 to -20.7), indicating an increase in the negative surface charge. The shift in zeta potential of the particles is indicative of the presence of negatively charged alginate layer on the GP surface. 

### Particle morphology

**Figure 2:**
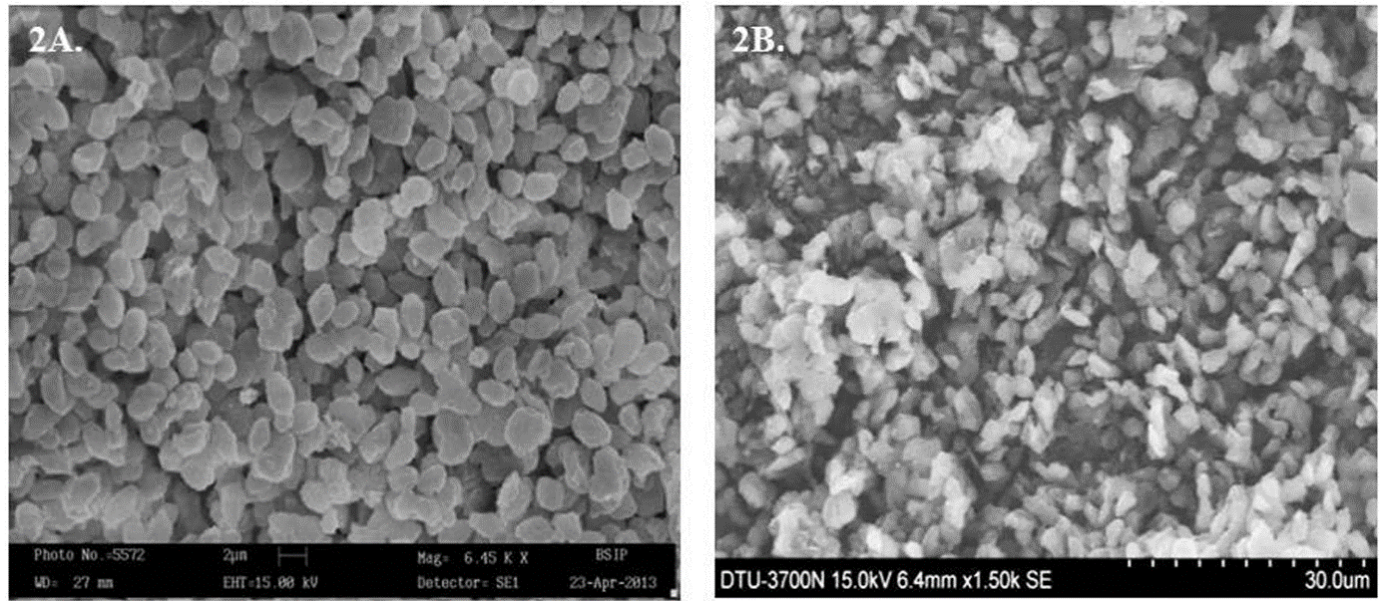
Scanning Electron Micrographs of (A) blank GP and (B) RB-loaded, alginate sealed GP

The SEM microphotographs showed the spray dried blank GP to be uniform, non-aggregated and nearly spherical to ellipsoidal (Figure 2A(Fig. 2)). The images also revealed the size of blank particles to be 2-4 µm, which was in good correlation with the size distribution data obtained by laser scattering, indicating that the GP were fairly dispersed. The incorporation of RB and alginate coating of these particles was seen to be accompanied with a change in particle shape. SEM revealed that while blank particles were nearly spherical, the drug loaded GP were more heterogenous, somewhat angular and ellipsoidal (Figure 2B(Fig. 2)). Some small GP appeared to aggregate with other particles after drug loading and alginate sealing. 

**Figure 3:**
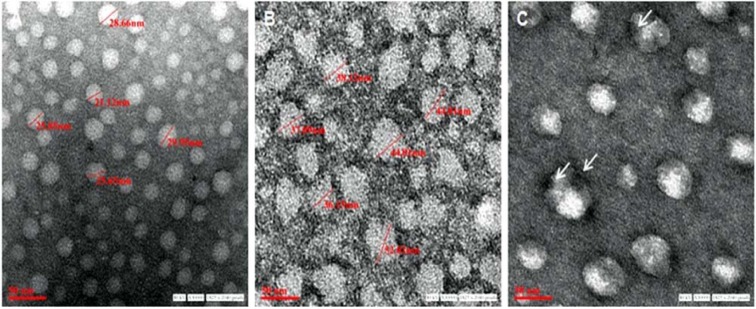
Transmission electron micrographs of (A) blank GP, (B) RB loaded unsealed GP, and (C) RB-loaded, alginate-sealed GP at 30,000 magnification. The red bar at bottom of images represents a 50 nm scale. The images reveal the porous nature of particles. Arrows in panel B point towards RB 'nanoprecipitates' or 'nanocrystals' formed within the GP pores in unsealed GP. The smooth and glistening surface visualized in Panel C, indicates the presence of an alginate layer sealing the drug loaded GP.

The TEM images of GP at all stages (blank GP, RB loaded GP and alginate sealed GP) show a highly porous structure (Figure 3(Fig. 3)). The electron micrograph shows clear, small, on an average ~25 nm pores in the blank GP (Figure 3A(Fig. 3)). In contrast, the images of drug loaded GP show a rough and speckled surface containing prominent drug 'nanoprecipitates' or 'nanocrystals' on the surface as well as filling the pore space (Figure 3B(Fig. 3)) , as previously suggested by Soto et al.*,* (2010[36]). The smooth textured, glistening surface in the micrograph of Figure 3C(Fig. 3) is indicative of the presence of an alginate layer coating on the GP surface.

### Structural studies of glucan in particles

#### Fourier Transform Infra-red (FTIR) analysis

**Figure 4:**
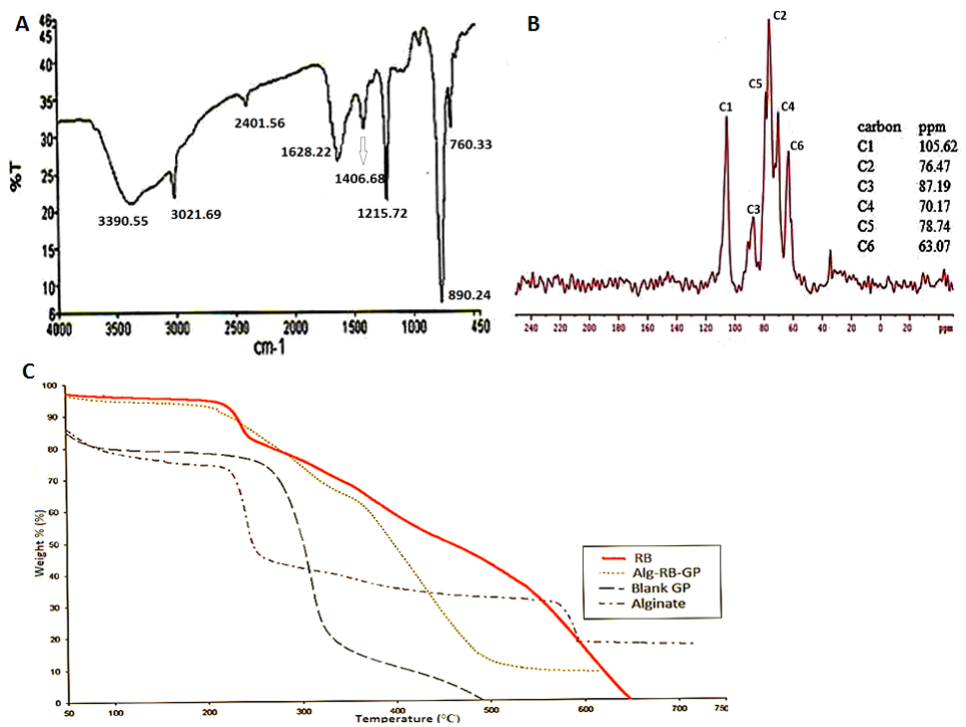
Structural and Thermal Stability analyses of GP. (A) FTIR spectrum of (spray dried) GP showing that particles were β-1,3 linked glucan. (B) Solid-State ^13^C-NMR Spectra of (spray dried) GP showing that the particulate glucan possesses a triple helical conformation. (C) Thermogravimetric curves of blank GP, Alginate, RB and RB loaded alginate sealed GP. Heating was done at a rate of 10 °C min^-1^ from 50 °C to 750 °C.

The prepared formulation was validated as particulate β-glucan by the FTIR analysis. The recorded FTIR spectra of blank GP formulation (Figure 4A(Fig. 4)), showed the typical signal pattern characteristic for β-1,3-D-glucan isolated from yeast (Hromadkova et al.*,* 2003[21]; Zechner-Krpan et al.*,* 2010[45]). The IR bands in the region of 928-1200 cm^-1^ (mainly due to C-C and C=O stretching vibrations in pyranoid rings) indicate the presence of polysaccharides as major component(s). Absorption peaks at 3390.55 cm^-1^ indicate O-H stretch, as the free hydroxyl groups absorb in the region of 3650-3500 cm^-1^., while the signals at 2401 and 1215.72 cm^-1^ indicate C-H stretch and CH_2_OH stretch respectively. The strong absorption peak at 890.24 cm^-1^ is characteristic of β-glycosidic bonds, i.e. (C1-H) deformation mode, and, therefore, indicates the presence of β-glucans. Absorption at 760 cm^-1^ implies the presence of α-glycosidic bond and could be explained by small concentrations of mannan, containing the above mentioned bond. The concentration of residual proteins in the β-glucan preparations with the presence of amide bands (amide I and amide II) vibrations of proteins at 1628 and 1406 cm^-1^, and almost similar results published by Hromadkova et al. (2003[21]).

### Solid state NMR spectroscopy

The high resolution ^13^C NMR spectra of the GP was recorded and compared with that of the reported spectra of native β-1,3-D-glucan in order to gain an insight into their primary and secondary structures in relation to their gel forming, physical and immunomodulatory properties (Falch et al.*,* 2000[11]).

#### Primary structure

The ^13^C NMR spectrum of particulate glucan in solid state (Figure 4B(Fig. 4)) was seen to be similar to that of native glucan reported elsewhere (Du et al.*,* 2012[9]; Saito et al.*,* 1987[30]). The chemical shifts from GP were observed at 105.62, 87.19, 78.74, 76.47, 70.17, and 63.07 ppm, which correspond to the C-1, C-3, C-5, C-2, C-4, and C-6 respectively. Thus, the NMR spectrum of the formulation verified the (1→3) β-D-glucan structure of GP. In addition, another small signal (C6^*^) was observed at 69.3 ppm, which is a characteristic peak due to β-1,6 branching (Du et al.*,* 2012[9]). The degree of branching (DB) of the water-insoluble yeast glucan is reported to be as low as 0.003 (Kim et al.*,* 2000[25]). Thus the NMR spectrum of GP confirmed the β-1,3 glucans structure with 1,6-linked side chain(s).

Since the C3 peak was found to resonate at 85.98 ppm (C-3b), a characteristic feature of the triple helix conformation (Saito et al.*,* 1987[30]), the prepared GP are expected to exhibit laminarin-type triple-helix conformation. On the contrary, the C3 peak of lentinan resonates at 89.6 ± 0.5 (C-3a) characteristic of the presence of curdlan-type single-helical form. Accordingly, the relative peak intensities between the C-3a and C3b peaks also suggest the presence of triple helix in GP. 

### Thermal stability analysis

The thermogravimetric curves (Figure 4C(Fig. 4)) were obtained for the GP-RB-Alg particles and their individual components, RB, blank GP and alginate. These curves show the variation of mass (%) as a function of temperature (° C). The TGA curve of sodium alginate suggests lower thermal stability than of glucan. 

Both sodium alginate and glucan showed similar type of weight loss pattern before decomposition. The decomposition temperature of sodium alginate and GP was lower than that of cross linked, drug loaded GP. However, RB thermogram shows sharp decomposition after melting and almost complete weight loss at ~ 650 °C. The TGA thermograms of the alginate sealed, RB loaded particles showed a decomposition and weight loss pattern similar to that of RB. However, it showed higher thermal stability than the blank GP and sodium alginate. 

The RB thermograms showed an endothermic peak at around 192 °C due to melting. There was no characteristic peak of RB in the thermogram of RB loaded GP-RB-Alg. These results indicated that RB was fairly dispersed in the GP. 

### Drug loading and encapsulation efficiency of the formulation

**Figure 5:**
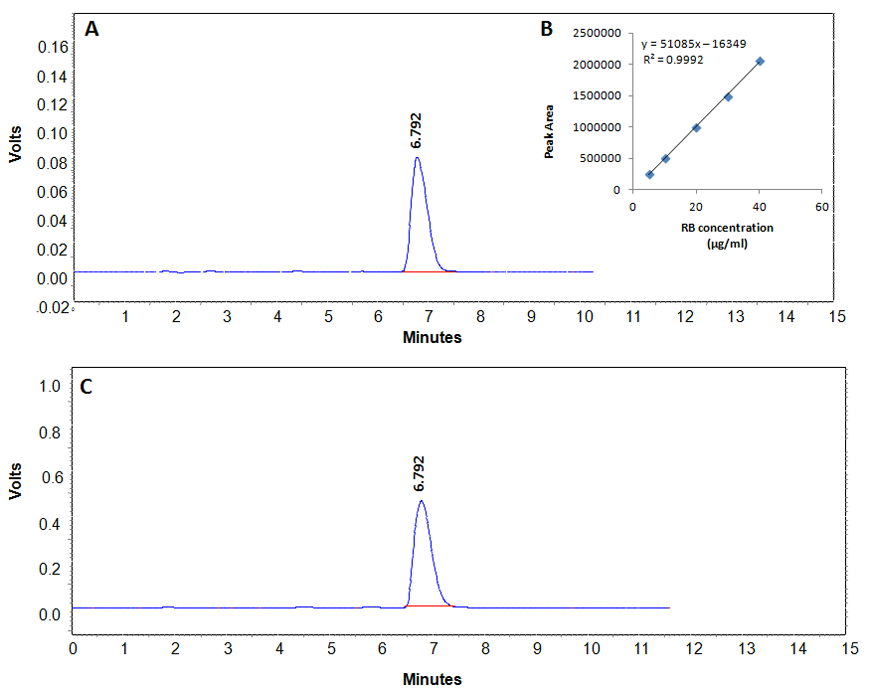
Quantitative analysis of RB. (A) HPLC chromatogram of RB in PBS buffer, (B) Standard curve of pure RB and (C) chromatogram of RB released from alginate sealed, RB-loaded GP sample.

HPLC chromatograms demonstrating RB peaks in standard and sample GP are illustrated in Figure 5A(Fig. 5) and 5C(Fig. 5), respectively. Standard calibration curves for RB analysis were linear in the concentration range of 5-40 µg/ml and showed regression coefficient (r^2^) > 0.995 (Figure 5B(Fig. 5)). The RB analysis by HPLC showed an increase in drug entrapment efficiency of GP formulations from 54.97 % to 80.74 % upon spray drying. The drug: polymer ratio of 1:1 was more or less maintained as such, since the drug loading was obtained up to ~40.5 %, indicating that the spray dried GP contain on an average, 40.5 % drug (RB) and 59.5 % polymer(s), including β-glucan and alginate.

Particle enumeration by flow cytometry revealed an average number of 1.68×10^8^ Rhodamine-labelled, blank GP (Rd-GP) per mg dry weight of the formulation. Upon drug loading and sealing, the particle number decreased to an average of 11.8 x 10^6^ particles per mg dry weight of the GP-RB-Alg formulation. Accordingly, each GP was found to contain on average, 34.3 pg RB. 

### In vitro release kinetics of loaded drug

**Figure 6:**
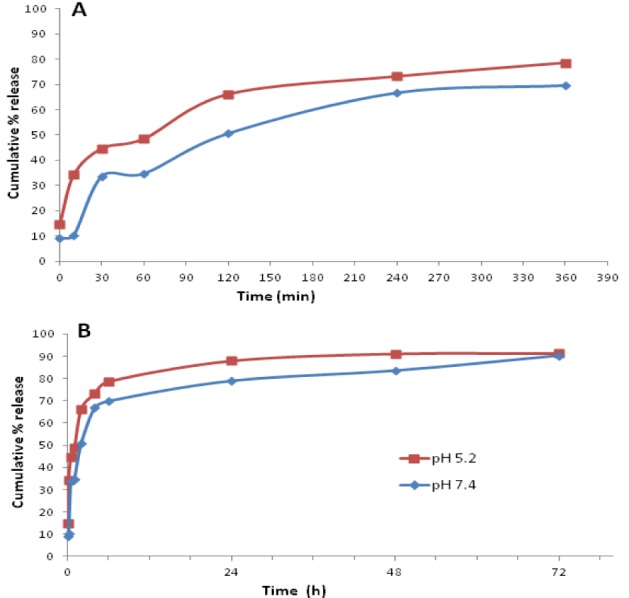
* In vitro* patterns of RB release from alginate sealed GP in cytosolic (phosphate buffer, pH 7.4) and phagolysosomal (acetate buffer, pH 5.2) conditions at 37 °C. (A) First 6 h analysis showed greater release of RB at pH 5.2 than at 7.4. (B) Overall RB release at cytosolic (pH 7.4) and phagolysosomal (pH 5.2) conditions up to 72 h

Upon internalization by Mϕ, the particles may be delivered either to classical, acidified phagosomes that undergo fusion with lysosomes; or may co-localize with bacteria in maturation-arrested phagosomes. The *in vitro* drug release from the GP-RB-Alg was thus studied at both cytosolic and lysosomal pH as shown in Figure 6(Fig. 6). 

The overall release behavior of RB from the alginate sealed GP was seen as biphasic with an initial burst effect, followed by a subsequent slow release as depicted in Figure 6A(Fig. 6). The first 10 min of drug release data showed 10.29 % of RB release at pH 7.4, and 34.26 % release at pH 5.2. The release pattern was nearly linear up to 4 h showing 66.86 % release at pH 7.4. During the same period, the release at pH 5.2 was 73.26 %. The curves tended to flatten out beyond this. 

The data indicated faster drug release at pH 5.2 than at 7.4 from the GP formulation (p < 0.05) up to 48 h. However, by 3 days, the cumulative amounts of RB release appeared to be almost same in both samples (p > 0.05), thereby indicating the possibility of a lack of pH-dependence over a longer time period. 

Various models of drug release were employed to analyse the drug release from particles (Table 1(Tab. 1)). The initial drug release up to 4 h seemed to be concentration dependent (first order kinetics), showing correlation coefficient (R^2^) value(s) of 0.953 at pH 7.4 and 0.891 at pH 5.2. The release pattern, however, showed best fit to the Higuchi model indicating a diffusion controlled drug release from the GP.

### In vitro cellular uptake and cytotoxicity of GP-RB-Alg 

**Figure 7:**
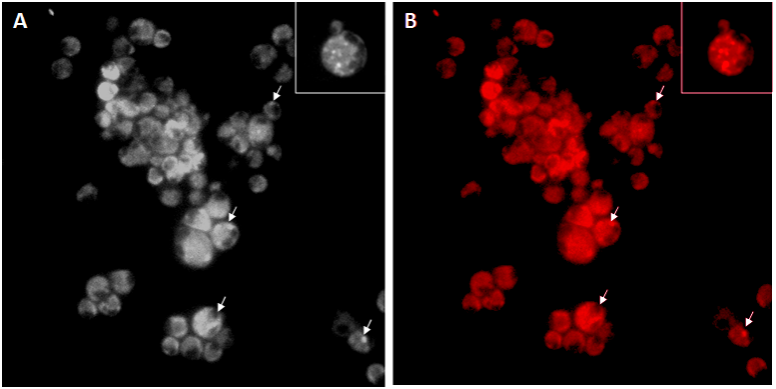
* In vitro* phagocytic uptake of Rhodamine B-labeled alginate sealed GP (Rd-GP) by J774 cells. *(A)* Bright field and (*B) *fluorescent micrograph of Rd-GP exposed macrophage at 20X magnification. White arrows show the internalized Rd-YDGP within macrophage after 5 min exposure

**Figure 8:**
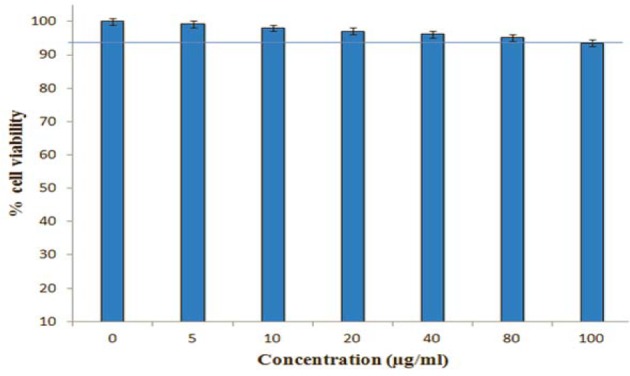
Effects of RB-GP on J774A.1 Macrophage cell viability. Cells were treated with various concentrations (0-100 μg/ml) of RB loaded GP for 24 h, after which their viability was detected by MTT assay. Data expressed as mean ± S.D from three independents experiments (*p < 0.05, ** < 0.01).

Microscopic examination of Mϕ exposed to the Rd-labeled GP-RB-Alg, revealed the presence of internalized particles emanating bright red fluorescence (Figure 7(Fig. 7)). The bright field images of Rd-labeled GP-exposed cells showed conspicuous granulation within the cytoplasm in comparison to the unexposed control cells, indicating uptake of fluorescently labeled GP by Mϕ within 5 min of exposure. To verify that experimental doses of Rifabutin loaded alginate sealed GP are not cytotoxic upon uptake by macrophages, we performed MTT viability assay against J774A.1. The RB loaded GP did not show cytotoxicity to J774 cells, upon 24 h exposure up to a concentration of 80 μg/ml (Figure 8(Fig. 8)). However, even upon of RB loaded GP exposure at 100 µg/mL concentrations the cells were about 90 % viable, suggesting these particles to be fairly biocompatible with the murine Mϕ cell line.

## Discussion

The smaller particle dimensions and higher uniformity (narrow size distribution) of the spray dried blank GP than the lyophilized particles, was in agreement with prior reports (Hunter et al.*,* 2002[22]; Zechner-Krpan et al.*,* 2010[45]). Homogeneous, small particles in the range of 0.3 to 10 µm are known to be readily phagocytosed by Mϕ and signal cellular activation. 

RB has been recommended by the WHO as a first-line agent for treatment of MDR-TB and is accepted as an effective replacement for Rif (Sirgel et al.*,* 2013[35]). Since the Rif-resistant/RB-susceptible isolates have been reported in much large numbers, RB has been suggested for the treatment MDR-TB and XDR-TB for positive treatment outcome in these patients. In addition, RB shows fewer drug-drug interactions and better tolerance by patients developing Rif-related adverse effects (Horne et al.*,* 2011[20]). It is more lipid soluble than Rif, resulting in more-extensive tissue uptake and was therefore, chosen for entrapment within the GP.

The HPLC studies revealed that despite a lower initial drug: polymer (1:1) ratio, the spray dried particles (GP2) had much higher drug loading as compared to the lyophilized particles (GP1), where an initially higher drug: polymer (2:1) ratio was taken. Since the spray dried blank GP (GP2) exhibited considerably improved characteristics such as small size, high uniformity, dispersion, yield, drug loading and drug encapsulation as compared to the lyophilized particles (GP1), the former were chosen for further experiments in this study.

The spray dried blank GP loaded with 50 % RB (1:1 drug: GP) showed an entrapment of 34.3 pg RB per GP. These values were much higher than those achieved by Soto et al. (2010[36]) who reported a drug content of 0.2 to 0.66 pg Rif per GP, prepared by loading 10 % and 33 % w/w Rif per GP, respectively (Soto et al.*,* 2010[36]). It is important to note that the Rifampicin-loaded GP reported earlier by Soto et al. (2010[36]) were prepared by yeast cell wall extraction followed by lyophilization and not spray drying. Sonication and spray drying leads to formation of micronized particles with increased porosity which leads to loading of large payload molecules within GP pores. The unexpectedly high drug loading obtained in the spray dried particles may be explained in terms of the microstructure of the porous blank GP formed by spray drying. We therefore, studied the particle morphology of GP at various stages of preparation by electron microscopy. In agreement with the reports by other investigators, the scanning electron micrographs (Figure 2(Fig. 2)) revealed a homogeneous, non-aggregated characteristic 'ellipsoidal'geometry of the spray-dried particles with an irregular surface (Yu et al.*,* 2015[43]). The β-glucan particle morphology and dimensions have earlier been shown to be influenced by the selection of drying method (Zechner-Krpan et al.*,* 2010[45]). Spray dried particulate glucan samples have been seen to preserve the ellipsoidal shape of yeast cells, and had lower viscosity than those dried by solvent exchange and lyophilization (Hromadkova et al.*,* 2003[21]). Additionally, the 'ellipsoidal' glucan particle geometry has been shown to be advantageous for Mϕ uptake (Garcia-Contreras et al.*,* 2008[12]). The Transmission Electron Micrographs (Figure 3(Fig. 3)) clearly reveals these particles to be highly porous, thereby permitting entrapment of drug molecules as 'nano-drug precipitates' or 'nanocrystals' of less than 50 nm within the GP (Soto et al.*,* 2010[36]). The presence of alginate layer on the GP surface was verified by the smooth, glistening porous surface visualized in the electron micrograph of sealed particles (Figure 3C(Fig. 3)) as well as by the negative changes in zeta potential (Figure 1C and D(Fig. 1)).

Since the glucan shell is formed prior to drug loading, it forms the structural framework of the GP-RB-Alg formulation. The spray dried particles are exposed to very high temperatures ranging from 90-170 °C (inlet and outlet air temperatures) and an atomizer pressure of 30-100 psi. The loading of appreciably higher magnitudes of drug within spray dried GP may be attributed to the efficient drying of the polymeric glucan resulting in formation of a more porous microstructure of the GP, as compared to that by lyophilization. Consequently, during drug loading, the 'drug-nanoprecipitates' get seated into the well-formed porous microstructure of the GP. The incubation of drug-loaded GP with alginate has been hypothesized to form a hydrogel matrix inside them, so as to physically entrap the payload drug, and seal the pores to retain the 'nanodrug precipitates' seated within them. The use of calcium chloride to cross-link the alginate layer on GP surface is known to further strengthen and stabilize the particles (Borges et al.*,* 2006[5]). 

The FT-IR analysis verified the primary β-1,3/1,6-glucan structure of spray dried particulate glucan, while the ^13^C NMR elucidated the triple helix conformation of β-glucan in these particles. This study is in agreement with a previous study which showed that the extraction procedure combined with spray-drying allowed the retention of the native β-glucan microstructure of the GP, with a triple-helix conformation (Zechner-Krpan et al.*,* 2010[45]). Another study by Hromadkova et al.*, *(2003[21]) reports the immunomodulatory activity of yeast-derived spray dried particulate glucan to be almost double of that with the particle samples dried by either solvent extraction or lyophilization (Hromadkova et al.*,* 2003[21]). 

The thermal characterization data indicated high thermal stability of the GP formulation. The GP-RB-Alg formulation was ~95 % stable at 170 °C temperature of the inlet air during spray drying. The polymer gel strength of alginate beads has been reported to depend on the concentration of calcium chloride used during preparation, the nature of the ionic salt used, sodium alginate concentration, and the presence of other polymers (Bajpai et al.*,* 2006[1]). The high thermal stability of alginate sealed GP might be attributed to the calcium alginate crosslinks, formed on the surface of particle shells, thereby probably lowering the elimination of small molecules like CO_2_, CO, or NaCl, and acting as an infusible support during the decomposition of GP formulation. Similar observation was also reported by Patil and Sawant (2009[28]) during DSC analysis of carvedilol drug encapsulated within alginate microspheres.

The initial burst release appears to come from the RB molecules adsorbed on the GP surface. The subsequent release was rather sustained and the loaded drug was released constantly and slowly up to ~24 h, whereafter it almost reached a plateau. The GP-RB-Alg formulations released ~90 % of their drug content at both the pH, over the first 72 h when tested *in vitro*. Soto et al. (2010[36]) demonstrated a release of 70-95 % of the loaded drug by GP over 17 h in PBS. They also demonstrated that the alginate or chitosan sealing retards drug release from GP and thereby, prolong the delivery of therapeutic molecules. 

Although alginate is more soluble at pH 7.4 than at pH 5.2, we observed a higher drug release at pH 5.2 than at pH 7.4 from GP-RB-Alg formulation. The observation may be attributed to the greater porosity and hydration of alginate matrix in acidic medium (Hodsdon et al.*,* 1995[19]) and a 16-fold higher RB solubility at pH 5.2 than at pH 7.4 (Marzolini et al.*,* 2001[26]). These particles may be expected to control drug release inside classical or maturation-arrested phagosomes over a few days to kill the replicating bacteria. 

Upon mathematical analysis, the data for* in vitro *drug release from GP-RB-Alg fitted the best to Higuchi's model (Table 1(Tab. 1)). The drug release data was also fitted to Korsmeyer-Peppas equation, which is often used to describe the drug release behaviour from polymeric systems when the mechanism is not well-known or when more than one type of release phenomena is involved. In the Korsmeyer-Peppas model equation (Table 1(Tab. 1)), 'k' is the rate constant, and 'n' is the release exponent providing information about the mechanism of drug release. At pH 5.2, the Korsmeyer-Peppas release exponent (n) value of < 0.45, indicated the drug release from the more porous and hydrated alginate matrix to be mediated by Fickian diffusion (Grassi and Grassi, 2005[14]). On the other hand, at pH 7.4, the value of 'n' (between 0.45 and 0.89) indicates an anomalous (non-fickian) transport mediated drug release from these particles (De Kee et al.*,* 2005[7]). It is suggested that at this pH, boundary conditions such as the presence of stagnant layer or a hydrogel on the release surface, results in an increased value of 'n'. The sharp front formed by penetration into gel matrix is assumed to slowly move into polymer linearly with time. The Korsmeyer-Peppas model has been reported to become identical with Higuchi model at nearly equal to 0.5 values of release exponent 'n' (Carriazo et al.*,* 2010[6]; Karewicz et al.*,* 2010[24]). This was observed in our studies showing drug release at pH 7.4 (Table 1(Tab. 1)), where the release exponent (n) obtained was nearly 0.5 and 'k' values obtained with the Higuchi and Korsmeyer-Peppas models were quite similar. 

Thus, the mechanism of drug release from GP appears to be as follows: Alginate matrices undergo hydration to create a gel layer, water penetrates the thin alginate layer sealing the GP, entering into polymeric matrix through small pores and channels; slowly dissolving and depleting the drug, which is released by diffusion from the pores into surrounding release medium. Leaching out of the drug from the pores and internal domains is expected to create empty spaces, permitting progressive hydration of the porous interior of alginate matrix.

The immunological activity of β-glucan is known to depend on particle dimensions and thus can be improved by reducing the GP size. The small size of particles, the triple helix configuration and a low degree of branching of the β-1,3-linked glucan polymer appears to advocate in favour of an effective immunostimulatory and therapeutic potential of this formulation. 

The *in vitro* uptake studies suggested that the GP were rapidly internalized by the mouse Mϕ cell line within 5 min of exposure. Our earlier, preliminary studies showed that GP uptake by the Mϕ started in less than 2 min of exposure. The 5 min exposure time was chosen to ensure sufficient and reproducible uptake. The GP were seen to retain their payloads for sufficient time to allow phagocytic uptake by Mϕ. 

Since each GP-RB-Alg has numerous 'drug nanoprecipitates' (as seen in the transmission electron micrographs), the uptake of even a single GP by macrophage would result in the intracellular delivery of a large number of drug nanoparticles. We therefore, expect the phagocytic uptake of GP-RB-Alg particles to result in high intracellular drug concentrations, as demonstrated in our previous studies with PLA based microparticles (Sharma et al.*,* 2001[33]).

Spray dried particles have been shown to be more effective at enhancing phagocytosis by peritoneal Mϕ than those prepared without spray drying (Hunter et al.*,* 2002[22]). GP are well known to bind dectin-1 receptors on Mϕ and DC (Batbayar et al.*,* 2012[2]), and are, therefore, capable of targeting and activating the innate immune system. Receptor-mediated endocytosis offers the potential to target selected cell types and enhance uptake of particulate material. Since GP are specifically recognized by Mϕ cells that express glucan receptors, they find potential application as Mϕ targeting formulations. This targeted delivery system has been recently shown to deliver loaded proteins efficiently and specifically to Mϕ without affecting non-phagocytic cells, such as NIH3T3, AD293, HeLa, and Caco-2 (Yu et al.*,* 2015[43]). This study therefore strongly advocates that GP can act as Mϕ targeting carriers to deliver the loaded drug molecules within these phagocytic cells known to harbor TB bacilli. 

The immune responses and anti-mycobacterial efficacy of these RB loaded GP formulations are yet to be determined. Nevertheless, it is encouraging to note that the GP-mediated Rif delivery to Mϕ, even at sub-MIC concentrations has been shown to enhance the antimicrobial effects of Rif (Soto et al.*,* 2010[36]). In line with this, the fact that our particles contain a high payload (~41 %) of RB, which reportedly has a much higher efficacy (8 times lower MIC range) than Rif, advocates for a much higher anti-mycobacterial potential of these particles, than that of previously reported GP containing only 10 % Rif. A slow-release drug delivery system also promises to reduce the frequency (days) and amount of drugs (doses) needed during conventional treatment. In this context, the *in vitro* release data from the GP-RB-Alg offers hope for the controlled release of the loaded antibiotics over a few days within the Mycobacteria infected Mϕ. Such drug containing GP, therefore, appear to be advantageous for TB chemotherapy, since they offer hope for improving patient compliance, targeting Mϕ-resident Mycobacteria, and perhaps also modulating the host's immunity towards a favorable therapeutic outcome. 

## Conclusion

This study clearly revealed that the glucan particles prepared by alkaline and acidic extraction followed by spray drying technology, exhibited considerably improved characteristics such as small size, uniformity, non-agglomeration tendency, high yield recovery and high magnitude of drug loading, as compared to those prepared without spray drying. Characterization of these particles showed a triple-helical conformation of β-1,3/1,6-linked glucans with a high thermal stability.

The spray drying technology permitted a higher drug loading than those prepared without spray drying, leading to achievement of magnitudes of (about 60 times) higher drug loading that reported by Soto et al.*,* (2010[36]). This is significant because low drug loading on the other hand, implies a need for repeated administration and inability to address the non-compliance problem and thus a risk for development of drug resistance. The drug release from alginate sealed GP was observed to be sustained and obeyed Higuchi's kinetics. The TEM images clearly reveal the presence of drug 'nanoprecipitates' or 'nanocrystals' filling the cavities of the GP. Thus, the phagocytic uptake of even a single GP by macrophage would result in the targeted intracellular delivery of a large number of drug nanoparticles. Thus, the spray dried GP-based-formulation technology holds promise for high drug loading and sustained release for sufficiently longer time periods, and thus has potential application as alternative dosage vehicles to target and deliver a large payload of anti-TB drug(s) to macrophage resident Mycobacteria. Our future studies are directed towards determining the immuno-stimulatory and antimicrobial potential of these GP formulations.

## Acknowledgements

The authors thank the U.P. Council of Science and Technology (UPCST) for financial support of this project (UP/CST/D-1382). Dr. Amit Misra and Dr. A.K. Dwivedi, Pharmaceutics division, CDRI, Lucknow, are gratefully acknowledged for their valuable suggestions during the particle preparation and characterization studies. Dr. Chauhan, IITR, Lucknow and Dr. Neeraj Sinha, CBMR Division, SGPGI, Lucknow, are also gratefully acknowledged for TEM imaging and Solid-State NMR studies. 

## Conflict of interest

The authors declare no conflict of interest and disclosures associated with the manuscript.

## Figures and Tables

**Table 1:**
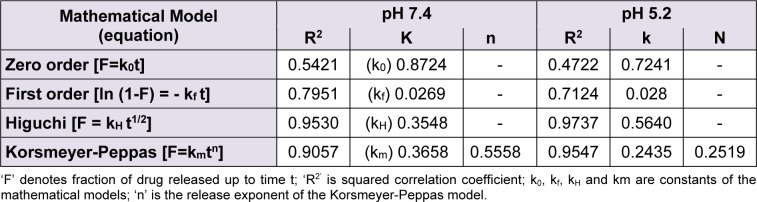
Squared correlation coefficient (R^2^) and coefficients obtained by mathematical model fitting to RB release data over 72 h
